# Ferumoxytol-enhanced magnetic resonance angiography for endovascular aortic repair surveillance in a patient after renal transplant

**DOI:** 10.1016/j.jvscit.2025.101898

**Published:** 2025-06-26

**Authors:** Richard Longfei Li, Daniel R. Ludwig, Anup S. Shetty, Vincent Mellnick, Brian G. Rubin

**Affiliations:** a Division of Vascular Surgery, WashU Medicine, St Louis, MO; b Malinkrodt Institute of Radiology, WashU Medicine, St Louis, MO

**Keywords:** EVAR, Ferumoxytol, MRI

## Abstract

Endovascular aneurysm repair requires lifelong imaging surveillance, typically with contrast-enhanced computed tomography imaging. This poses risks to patients with end-stage renal disease. Ferumoxytol, a superparamagnetic iron-based nanoparticle with minimal nephrotoxicity, has emerged as an alternative contrast agent for magnetic resonance angiography in patients with renal impairment. We present a case of a patient with a failing renal transplant who underwent ferumoxytol-enhanced magnetic resonance angiography to further characterize an indeterminant etiology of continued aneurysm sac expansion to 10 cm, avoiding iodinated and gadolinium-based contrast while achieving high-quality imaging for endoleak detection.

Endovascular aneurysm repair (EVAR) is a minimally invasive treatment option for abdominal aortic aneurysm (AAA) repair. Surveillance imaging is required to monitor for complications such as endoleaks, graft migration, and aneurysm sac expansion.^1^ Although computed tomography angiography (CTA) remains the gold standard, 27% of patients with an estimated glomular filtration rate <30 mL/min/1.73 m^2^ develop contrast-induced nephropathy following iodinated contrast exposure.^2^ Magnetic resonance angiography (MRA) with gadolinium-based contrast agents (GBCAs) is a viable alternative.^3^ Although GBCAs are not nephrotoxic at doses used for MRA, they are associated with a less than 0.07% risk of nephrogenic systemic fibrosis (NSF) in patients with renal insufficiency.^4^ Given the high prevalence of end-stage renal disease (ESRD) in the vascular patient population and the serious nature of NSF,^5^^,^^6^ alternative imaging strategies are needed to ensure safe and effective post-EVAR surveillance.

Ferumoxytol (Feraheme, Covis Pharma) is an ultrasmall superparamagnetic iron oxide particle. Unlike iodinated and gadolinium-based agents, ferumoxytol is not nephrotoxic and has not been associated with NSF.^7^ United States Food and Drug Administration-approved in 2009 for iron deficiency anemia, the large molecular size of ferumoxytol confers a prolonged intravascular half-life, with eventual clearance by the mononuclear phagocyte system. These properties enable its off-label use as a contrast agent for magnetic resonance imaging (MRI)/MRA, providing excellent blood-pool imaging.

This case highlights the application of ferumoxytol-enhanced MRA (Fe-MRA) in a patient with a failing renal allograft to identify a type II endoleak not previously seen on CTA. The patient provided his consent for the use of his images and clinical course for this report.

## Case report

**Fig 1:**
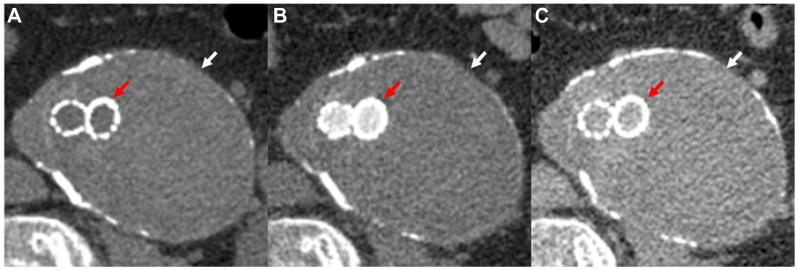
Computed tomography angiography (CTA) with noncontrast **(A)**, arterial **(B)**, and venous **(C)** phases demonstrating a large abdominal aortic aneurysm (AAA) measuring approximately 7.5 × 9 cm (*white arrow*) with a bifurcated endovascular graft in place (*red arrow*). There was no clear evidence of endoleak.

**Fig 2:**
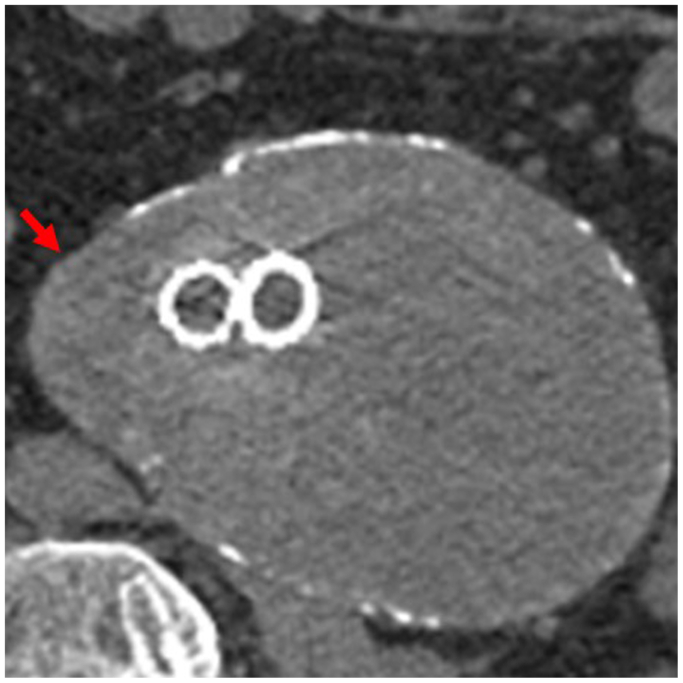
Noncontrast surveillance computed tomography (CT) scan demonstrating sac expansion to 8.7 × 10.2 cm with a sac volume of 516 mL. In this study, there was increased focal outpouching of the right aspect of the aneurysm sac (*red arrow*) compared with the prior study, concerning for instability.

A 66-year-old man with coronary artery disease and ESRD postrenal transplant in 1999 underwent EVAR with a Gore Excluder in 2018 for a 5.5-cm AAA. He was initially lost to follow-up, but during his re-transplantation workup, he was referred to our institution in 2023. where his aneurysm sac was found to have expanded to 7.5 × 9 cm. CTA at that time failed to identify a source for his continued sac expansion (Fig 1). In 2024, the patient was treated for a presumed type IV endoleak, and his EVAR was relined with a 23 mm × 3.3 cm Gore Aortic Extender and two Gore Excluder limbs (16.5 × 9.5 cm and 14.5 × 10 cm). In 2018, the patient’s estimated glomular filtration rate was 45 mL/min/1.73 m^2^, which declined to 18 mL/min/1.73 m^2^ at the time of his Fe-MRA. Given his failing allograft and continued AAA sac expansion to 8.4 × 11 cm on noncontrast surveillance CT (Fig 2), we proceeded with Fe-MRA to further evaluate for an endoleak.

### Imaging technique

The study was conducted on a Siemens MAGNETOM Sola (1.5 Tesla). Sequences included T1-weighted time-resolved angiography with interleaved stochastic trajectories (TWIST) acquired in the coronal plane, and T1-weighted axial and transverse pre-and postcontrast breath-held-three-dimensional (3D) fast low-angle shot (FLASH) MRA. Ferumoxytol was administered under nursing supervision as a 1 mL/second bolus injection in the scanner, totaling 3 mg/kg diluted to 50 mL.

### Imaging analysis

**Fig 3:**
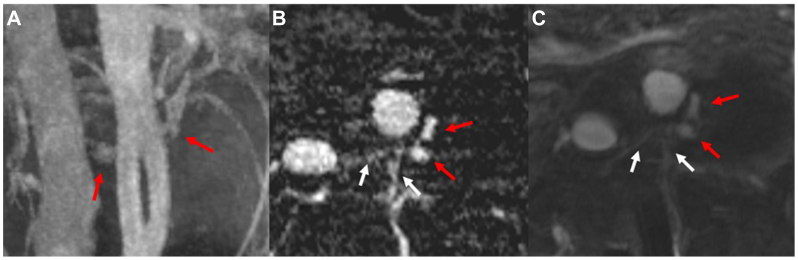
Magnetic resonance angiography (MRA) characterization of bilateral L2 lumbar endoleaks. **(A)** A maximum-intensity projection coronal time-resolved angiography with interleaved stochastic trajectories (TWIST) subtraction image demonstrating a patent bifurcated endograft with endoleaks just lateral to the flow divider; **(B)** A TWIST subtraction image reformatted in the axial plane; and **(C)** An axial postcontrast fast low angle shot (FLASH) three-dimensional (3D) image. *White arrows* indicate patent L2 lumbar arteries, whereas *red arrows* highlight evidence of perfusion to the aneurysm sac consistent with a type II endoleak.

Postprocessed images (ie, maximum-intensity projection and subtraction images) were generated on the scanner. Images were reviewed and interpreted on the clinical PACS (Sectra PACS, Sectra Medical). Time-resolved MRA images after ferumoxytol administration demonstrated a type II endoleak arising from the paired lumbar arteries at the level of L2 (Fig 3, *A*). Axial reformatted TWIST subtraction images acquired during a late arterial phase and axial FLASH 3D postcontrast images best demonstrate the paired lumbar arteries perfusing the left posterolateral aspect of the aneurysm sac adjacent to the proximal portion of the device (Fig 3, *B* and *C*). Time-resolved MRA imaging also provided insight into the flow pattern and volume of the feeding vessels (Supplementary Video, online only).

At present, the patient is undergoing further workup for more pressing medical issues and has opted to continue with surveillance.

## Discussion

Surveillance post-EVAR remains critical. The incidence of endoleaks by types I to III are estimated to be approximately 10%, 25%, and 4%, respectively, with types IV and V becoming exceedingly rare in modern endovascular designs.^8^ In the setting of reduced kidney function, available options at our institution include noncontrast CT scans, low-dose dual-energy contrast-enhanced CT, MRI with GBCAs, angiography, and duplex ultrasound. These modalities are either unable to delineate the source of sac perfusion or are invasive. Dual energy contrast-enhanced CT has the benefit of lower contrast burden but has higher noise levels, inferior image quality, and worse diagnostic accuracy compared with single energy.^9^ Duplex ultrasound is effective but limited by operator variability, body habitus, and bowel gas. Ultrasound might have identified sac perfusion but not the specific feeding vessels, proximal seal, or stent migration.^10^

Ferumoxytol carries no known risk for NSF with minimal long-term tissue retention.^11^ The United States Food and Drug Administration has issued a black box warning regarding the risk of serious hypersensitivity reactions (anaphylaxis) following intravenous injection, and has identified bolus injection of undiluted ferumoxtyol as a primary risk factor.^12^ To mitigate this risk, MRI/MRA imaging uses heavily diluted concentrations of ferumoxytol as an intravenous infusion or bolus injection. In cases where bolus injection is needed to address the clinical question, such as in investigating vascular pathology and contrast extravasation, bolus injection of diluted ferumoxtyol is performed under direct nursing supervision, with continuous monitoring of vital signs during MRI scanning.

Ferumoxytol’s large molecular size and surface coating prolong its intravascular half-life, making it well-suited for vascular imaging and endoleak detection.^13^ These properties facilitate the identification of subtle endoleaks, with blood-pool Fe-MRA capable of detecting leak rates as low as <0.1 mL/min.^14^ Several studies have shown that Fe-MRA may have a higher sensitivity for endoleak detection compared with traditional CTA.^15^^,^^16^ A review by Habets et al found that Fe-MRA correctly identified nearly twice as many endoleaks as CTA.^17^ To highlight the safety and efficacy of this modality, a study by Hubbard et al investigated a series of 21 patients and were able to successfully identify and characterize type IA, IB, and II endoleaks with no reported adverse events.^18^

Fe-MRA is not without drawbacks. Although CTA and MRA are similarly priced ($773.28 vs $760.88^19^), Fe-MRA is labor-intensive, requires nursing supervision, and uses a contrast agent costing approximately $70/mL,^19^ compared with $10/mL for Gadovist.^20^ These factors may limit widespread adoption despite its diagnostic advantages.

This case demonstrates the successful identification of a persistent type II endoleak in a patient with ESRD and a poorly functioning allograft. In line with prior literature, Fe-MRA enabled detection of the endoleak without compromising renal function. Our institution is actively incorporating Fe-MRA into its surveillance algorithm for patients with indeterminate sac expansion or compromised renal function. Further studies are needed to directly compare gadolinium-enhanced MRA, Fe-MRA, and contrast-enhanced ultrasound for endoleak detection in this high-risk population.

## Conclusion

Fe-MRA offers a safe, effective alternative for EVAR surveillance in patients with renal insufficiency or nondiagnostic CTA, addressing critical limitations of CTA and gadolinium-based alternatives. Ferumoxytol should be considered as a viable imaging modality in EVAR surveillance, especially in patients with advanced renal disease or indeterminate sac expansion on CTA.

## Funding

None.

## Disclosures

None.

## Appendix (online only)

Supplementary Video (online only) Coronal maximum intensity projection time-resolved angiography subtraction imaging demonstrating real-time filling of L2 lumbar arteries into the aneurysm sac.
